# Optimal administration of bronchodilators with valved holding chambers in preschool children: a review of literature

**DOI:** 10.1007/s00431-021-04074-3

**Published:** 2021-04-20

**Authors:** Péter Csonka, Terhi Tapiainen, Mika J. Mäkelä, Lauri Lehtimäki

**Affiliations:** 1 Centre for Child Health Research, Tampere University and Tampere University Hospital, Kalevantie 4, 33014 Tampereen yliopisto, Tampere, Finland; 2 Terveystalo Healthcare, Tampere, Finland; 3 Department of Pediatrics and Adolescent Medicine, Oulu University Hospital and PEDEGO Research Unit, University of Oulu, Oulu, Finland; 4 Skin and Allergy Hospital, Helsinki University Hospital and University of Helsinki, Helsinki, Finland; 5 Allergy Centre, Tampere University Hospital, Tampere, Finland; 6 Faculty of Medicine and Health Technology, Tampere University, Tampere, Finland

**Keywords:** Bronchodilators, Valved holding chambers, Preschool children, Spacer, Acute, Management, Asthma, Wheezing, Emergency treatment, Inhalation therapy, Guidelines, Drug delivery

## Abstract

Our aim was to synthesize the published literature on factors that potentially affect the delivery of bronchodilators using valved holding chambers (VHC) in preschool children. We also aimed to identify those attributes that are not yet incorporated or clearly stated in the guidelines and those topics that are still lacking sufficient data. There is strong evidence supporting several recommendations in current guidelines. Based on present knowledge, bronchodilators should be delivered by VHC administering each puff separately. Face mask should be omitted as soon as the child can hold the mouthpiece of the VHC tightly between the lips and teeth. Based on the review, we suggest adding a specific note to current guidelines about the effect of chamber volume and the impact of co-operation during drug administration. Calming the child and securing a tight face-to-mask seal is critical for successful drug delivery. There is not enough evidence to make specific recommendations on the most reliable VHC and face mask for children. There is an urgent need for studies that evaluate and compare the effectiveness of VHCs in various clinical settings in wide age-groups and respiratory patterns. In addition, there is insufficient data on ideal chamber volume, material, and effective antistatic treatment.

**Table:** 

**What is Known:** • *Valved holding chambers (VHC) should not be considered interchangeable when used with pressurized metered dose inhalers (pMDI)*. • *Drug delivery is influenced by VHC volume, aerodynamic and electrostatic properties; mask fit; respiratory pattern and co-operation during inhalation; and the number of puffs actuated*. **What is New:** • *The impact of co-operation, VHC volume, and good mask-to-face fit during drug inhalation is not stressed enough in the guidelines*. • *Studies are urgently needed to evaluate the effectiveness of different VHCs in various clinical settings focusing on VHC electrostatic properties, respiratory patters, face masks, and ideal pMDI+VHC combinations*.

**What is Known:**

• *Valved holding chambers (VHC) should not be considered interchangeable when used with pressurized metered dose inhalers (pMDI)*.

• *Drug delivery is influenced by VHC volume, aerodynamic and electrostatic properties; mask fit; respiratory pattern and co-operation during inhalation; and the number of puffs actuated*.

**What is New:**

• *The impact of co-operation, VHC volume, and good mask-to-face fit during drug inhalation is not stressed enough in the guidelines*.

• *Studies are urgently needed to evaluate the effectiveness of different VHCs in various clinical settings focusing on VHC electrostatic properties, respiratory patters, face masks, and ideal pMDI+VHC combinations*.

## Introduction

Acute bronchoconstriction and viral wheezing account for approximately 10% of the emergency room (ER) visits in children resulting in hospitalization in 30–50% of cases [1, 2]. Bronchodilators administered by pressurized metered dose inhalers (pMDI) via valved holding chambers (VHC) are clinically at least as effective as nebulizers [2–7]. Although most current guidelines recommend the use of VHC for the delivery of bronchodilators in acute bronchial obstruction in young children [8–14], nebulizers are still widely used. In the era of coronavirus disease 2019 (COVID-19) pandemic, there is considerable concern about the potential risk of transmission the virus in the form of aerosolized respiratory droplets by nebulizers [15, 16], although preschool children are not the main spreaders of COVID-19. Consequently, the use of metered-dose inhalers (MDIs) have increased considerably as an alternative to nebulized therapy.

In clinical practice, the most reliable and effective VHC delivery system—with and without face masks—and optimal dosing regimen of bronchodilators should be used. However, each VHC has its own unique features related to material, electrostatic and aerodynamic properties, volume, dead space, and valve design. Furthermore, the face mask fit and crying during inhalation administration may affect the treatment efficacy.

Our aim was to synthesize the published literature on factors that potentially affect the delivery of bronchodilators using valved holding chambers (VHC) in preschool children. We also aimed to identify those attributes with sufficient evidence that are not yet incorporated or clearly stated in the guidelines, and those topics that are still lacking sufficient data.

## Review of the literature

We searched the following electronic databases up to October 25, 2020, to capture systematic reviews and individual trials: Medline (OVID), US National Library of Medicine PubMed, Cochrane Database of Systematic Reviews, Duodecim Terveysportti database in Finland, Google Scholar, and Google internet search engine. The following search terms were used: *nebulizer, nebuliser*, *spacer* or *valved holding chamber* in combination with any of the following: *antistatic, static, washing, priming, mask, face mask, facemask, seal, fit, wheezing, asthma, salbutamol, albuterol, bronchodilator, beta-sympathomimetic(s) number of puffs, guideline,* and *review.* The search was limited to English and Finnish language publications. In addition to searching the electronic databases, we screened the reference lists from the already identified studies, review articles, and guidelines for any additional studies. There were 3221 records with abstract identified. After the duplicates were removed, 3092 records were screened, and 2928 records were excluded. Altogether 124 full-text articles were assessed for eligibility, and 66 full-text articles were excluded. Finally, 58 studies were included in the synthesis.

### Delivered dose of bronchodilators with different types of VHC

Each VHC has its own unique features related to material, electrostatic and aerodynamic characteristics, volume, dead space, and valve design [17–21] (Table 1). Thus, certain combinations of pMDI+VHC may result in marked differences in dose output [18, 19, 21–27]. For example, using tidal volume Vt 50 ml and RR 30/min Mitchell et al. [24] found that one of the three VHCs investigated (Space Chamber, Vent-170 and Child AeroChamber) yielded undetectable levels of salbutamol compared to 37 μg filter dose from another device. A recent study observed markedly better salbutamol delivery with Optiochamber Diamond compared to Babyhaler especially with Vt below 150 mL [28]. In addition, similar devices from different brands may have up to 20-fold differences in drug delivery capacity in experimental *in vitro* models [17, 20].

**Table 1:** Characteristics of some valved holding chambers (VHC) on the market worldwide. Each device has its own range of face masks. Disposable cardboard VHCs are not compatible with any mask. Face masks vary considerably in volume (40–100 ml), softness and fit. DispoZABLE spacer has no valve (not a VHC) but is marketed also for children

	**VHC volume**	**Chamber material** and static charge specified by the manufacturer*	**Valve characteristics and additional features**
**A2A Spacer (A2A)**	210 mL	ABS, antistatic	Internal circular unidirectional silicon valve, aerosol passes through the valve centrally
**Aerochamber plus**	149 mL	ABS, antistatic	Internal circular unidirectional silicon valve, aerosol passes at the valve periphery, top outside inspiratory indicator
**Babyhaler**	350 mL	Polycarbonate, non-electrostatic	Internal circular unidirectional silicon valve hinged centrally, aerosol passes at the valve periphery, additional top outside valve
**Compact Space Chamber Plus**	160 mL	ABS, antistatic	Internal silicon valve with central cross shaped opening
**DispoZABLE spacer**	N/A	Cardboard	No valve. No Mask.
**InspiraChamber**	N/A	ABS, antistatic	Internal cone shaped unidirectional silicon valve, aerosol passes through the valve centrally
**LiteAire Disposable**	N/A	Cardboard	Dual plastic sheet valves. Disposable spacer. No mask.
**Livingstone Disposable Cardboard Asthma Spacer**	N/A	Cardboard	Dual plastic sheet valves. Disposable spacer. No mask.
**Optichamber diamond**	140 mL	ABS, antistatic	Internal cone shaped unidirectional silicon valve, aerosol passes through the valve centrally, additional top outside expiratory valve
**ProChamber**	145 mL	ABS, antistatic	Internal cone shaped unidirectional silicon valve, aerosol passes through the valve centrally
**Rossmax Aero Spacer**	175 mL	ABS, antistatic	Internal circular unidirectional silicon valve, aerosol passes through the valve centrally
**Tipshaler**	N/A	N/A	Internal cone shaped unidirectional silicon valve, aerosol passes through the valve centrally
**Volumatic**	750 mL	Polycarbonate, non-electrostatic	Internal rigid circular plastic valve, aerosol passes at the valve periphery. No mask.
**Vortex**	194 mL	Aluminum, reduced static charge	Internal cone shaped unidirectional silicon valve, aerosol passes through the valve centrally

*Manufacturers use several different terms: antistatic, non-electrostatic or reduced static charge. All non-conductive materials (such as ABS, polycarbonate, and cardboard) are prone to accumulate electrostatic charge.

ABS, acrylonitrile butadiene styrene

N/A, not available

The review of the literature showed that VHCs are not interchangeable and recommended drug doses may have to be adjusted according to the properties of each pMDI+VHC combination. However, there is insufficient evidence regarding the clinically most effective VHC. This is reflected in the current lack of standardization and variations in the use of these devices. There are no specific recommendations in the guidelines about VHC models (Table 2).

**Table 2:** Overview of the current treatment guidelines of optimal administration of bronchodilators with valved hold chambers (VHCs) in preschool children and further recommendations by the current review. *pMDI* pressurized metered dose inhalers

Guideline	pMDI+VHC or nebulizer	Recommendation concerning the choice of VHC	Recommendation concerning the face mask usage and seal	Notes on cooperation	Number of puffs to be used at a time	Recommendation concerning VHC handling
GINA [9]	pMDI+VHC	Indicates that young children can use spacers of all sizes, but a lower volume spacer (< 350 mL) is advantageous in very young children.	Instructs that a tightly fitting face mask should be used for children under 4 years. Face mask should be switched to mouthpiece as soon as children are able to demonstrate good technique.	No specific notes.	One	States that to reduce static charge, plastic VHC should be pre-washed with detergent and air-dried to be ready for immediate use.
Australia [10]	pMDI+VHC	Mentions that there are different types of VHC but does not recommend any one specific VHC for acute treatment.	Instructs that a well-fitted mask should be used for small children who cannot form a tight seal with their lips around the spacer mouthpiece.	Remarks that babies are unlikely to inhale enough medicine while crying and there should be extra effort to calm the children down in order to ensure adequate therapeutic effect.	One	States that to reduce electrostatic charge standard plastic VHC should be pre-washed with detergent. Treatment to reduce electrostatic charge is not necessary for cardboard and polyurethane/antistatic polymer spacers.
USA [13]	pMDI+VHC	Indicates that due to the significant variation found between the performance of specific VHCs and pMDIs, it may be preferable to use the same combination of pMDI+VHC reported in the individual drug study to achieve comparable results.	Instructs that a tightly fitting face mask should be used for children under 4 years and for those who are unable to use mouthpiece.	No specific notes.	One	Instructs to use antistatic VHCs or to rinse static plastic VHCs with dilute household detergents to enhance delivery to lungs and efficacy.
UK [11]	pMDI+VHC	Indicates that each VHC should be compatible with the pMDI being used and that the change in VHC may alter effective dose delivered.	Instructs that a face mask should be used for those who are unable to use mouthpiece. Does not mention about the face mask fit.	No specific notes.	One	Instructs that VHCs should be cleaned monthly rather than weekly as per manufacturer’s recommendations or performance is adversely affected.
Canada [12]	pMDI+VHC	No specific recommendations.	Indicates that for children 1-3 years of age, a VHC with a correctly sized facemask is preferred. Does not mention about the face mask fit.	No specific notes.	Not mentioned.	No specific recommendations.
Finland [8, 14]	pMDI+VHC	No specific recommendations.	Instructs that a face mask should be used for children under 3 years. Does not mention about the face mask fit.	No specific notes.	Not mentioned.	No specific recommendations.
Current evidence and further recommendations by the current review	Evidence supports using pMDI+VHC instead of a nebulizer. Current guidelines are in line with the evidence.	There are considerable differences in drug delivery between VHCs, but their clinical implications are not known. Guidelines should note that the dose output of different VHC models vary significantly and VHCs may not be interchangeable. Future studies are needed to assess the clinical effect of these differences.	Face mask should be used in children who are unable to hold the VHC’s mouthpiece between the lips. Tight fit and good seal between the mask and face is essential for drug delivery. Guidelines are in line with the evidence in recommending the use of face masks. Guidelines should also emphasize that to ensure optimal drug delivery good face mask fit should be routinely check before and during drug administration. Face mask should be omitted as soon as the child is able to hold the mouthpiece tightly between the lips.	Crying and poor co-operation of the child may significantly decrease pulmonary drug delivery. Guidelines should indicate that crying and poor co-operation during inhalation may significantly reduce pulmonary drug delivery. Calming the child is important for optimal drug delivery.	Better drug delivery can be achieved by inhaling each dose separately. Most of the guidelines are in line with the evidence, but this should be noted in every guideline.	Different chamber materials have variable electrostatic properties and VHC handling may significantly affect drug delivery. Future sponsor-independent studies are needed to evaluate the clinical impact of the electrostatic effect and antistatic treatment of VHCs.

### Impact of VHC volume on the output of bronchodilators

In children, larger volume VHCs (> 200 mL) may result in a lower salbutamol output compared with smaller VHCs (< 200 mL) [17, 22, 27, 29]. In large volume VHCs, the aerosol concentration and particle impaction are less than in a smaller volume VHC. This can result in lower inhaled doses at a decreased Vt but higher doses when the VHC can be emptied faster with a larger Vt [29–33]. During bronchoconstriction, Vt decreases and RR increases, and asthmatic patients have a higher range of variation in PIFR compared to healthy subjects [34]. Operation of VHCs under suboptimal conditions is clinically important since children below school age can have inspiratory flow rates as low as 2 L/min [34]. Only the GINA guidelines [9] indicate that using spacers with less than 350-mL chamber volume is advantageous in very young children (Table 2).

### Effect of the respiratory pattern on drug delivery through VHCs

Drug delivery through VHCs is shown to be dependent on the breathing pattern and respiratory characteristics of the patients, both *in vivo* and in experimental models [17, 29–33, 35]. Inspiratory flow rate (PIFR) is linearly correlated with Vt [29, 34] and asthmatic patients have a wider range of PIFR compared with healthy subjects [34].

Increasing Vt is associated with increasing drug delivery. Chavez and colleagues [36] found in an in vitro study using AeroChamber Max® that when gradually increasing the Vt from 36 to 290 ml, the filter dose of albuterol increased in a logarithmic fashion with both RRs of 12 and 24 L/min.

During bronchoconstriction, Vt decreases and respiratory rate (RR) increases. The drug delivery capacity seems to be sensitive to shallow and rapid respiratory pattern related to airway obstruction [17, 22]. For example, with Vt of 30–50 ml and RR of 25–30/min, some VHCs yielded undetectable in vitro filter doses of salbutamol [22, 24].

Mitchell *et al*. [24] observed marked differences in the effect of breathing patterns on drug delivery between three VHC devices. At low tidal volume (50 ml), no salbutamol was delivered by either the Vent 170 or Space Chamber^TM^, whereas the unit dose from the AeroChamber® was 39.7 ± 1.6 μg. The Vent-170 and Spacer Chamber^TM^ delivered measurable doses for salbutamol when the tidal volume was increased to 100 ml and to 200 ml; however, the corresponding doses available from the AeroChamber® was always significantly greater [24].

We did not find clinical data comparing different VHCs in small children with severe bronchial obstruction having very low Vt and high RR. The effect of variable respiratory pattern by age and during bronchoconstriction is not specifically addressed the guidelines.

### Effect of face mask, face mask seal, and patient co-operation on drug delivery with VHCs

Face masks are used to facilitate drug delivery in young children who are incapable of holding a mouthpiece between the lips and teeth [9]. However, drug delivery is lower when face mask is used [17, 36]. Children older than 3–4 years of age are generally able to use VHC without masks [9], and most guidelines recommend that face mask should be omitted as soon as children are able to demonstrate good technique [8–11, 13].

A good seal with a minimal leak around the nose, cheeks, and mouth will ensure inspiration through the VHC and prevents ingress of ambient air between the mask and face [37, 38]. Several in vitro studies have shown that leakage due to the lack of a tight mask-to-face seal has a large impact on aerosol delivery [17, 20, 37, 39–41]. Even a minor air leak between the face and the mask can drastically reduce aerosol delivery and depending on the location and size of the mask leak, and drug delivery can drop almost to an undetectable level [41]. According to Esposito-Festen et al. [41], the lung dose seems to decrease more rapidly when the mask leak is located close to the nose relative to a leak near the chin. The overall design, volume, and degree of adaptability of the face mask are important factors that influence the fit and mask-to-face seal as well as the physical dead space of the mask [32]. When pressed against the face, pressure causes compression of the mask, tightening the contact and reducing the actual dead space of the mask [42, 43]. However, with increasing pressure against the face, children are more likely to cry which again decreases the inhaled dose [44–46].

When aerosol is inhaled during crying, lung deposition decreases significantly [46, 47]. In a study by Erzinger at al. [45], lung deposition of radiolabeled drug relative to the total nominal dose was 0.2–0.3% in children who inhaled with a non-tightly fitted face mask. The deposition was 0.6–1.4% in screaming children with a tightly fitted face mask and 4.8–8.2% in patients inhaling quietly. Approximately 2–5 breaths per actuation seem to be enough to empty the VHC [23, 25, 48]. However, the optimal number of breaths required to empty the VHC depends on the child’s Vt, the volume of the VHC and valve dead space.

Guidelines are in line with the evidence in recommending the use of face masks for younger children, but they do not fully endorse good face mask fit or the importance of calming the child to improve co-operation and drug delivery (Table 2).

### Impact of taking each puff separately vs. multiple puffs simultaneously

Clark and Lipworth [49] measured higher plasma salbutamol levels per actuation and greater systemic responses after inhaling each puff separately from the Volumatic VHC (750 mL) compared with either inhaling multiple puffs simultaneously or taking single puffs with delayed inhalation. Also using the Volumatic, Barry and O’Callaghan [50] found that compared with single actuation, double actuation for one inhalation decreased drug recovery by 22% per 100 μg actuation and quintuple actuation decreased it by 62% per 100 μg actuation. In the study by Wildhaber et al. [51], the total amount of salbutamol delivery from Babyhaler (350 mL) was reduced by 17% per actuation for two puffs at a time and by 22% per actuation for five puffs at a time. In the same study the differences in drug delivery for multiple actuations from the antistatically treated Babyhaler and the antistatic Nebuchamber were less pronounced but still statistically significant. The effect of multiple actuations may vary depending on the VHC brand. In the study by Csonka and Lehtimäki [52], four out of the six VHCs examined showed significantly poorer performance per actuation with two puffs as opposed to one puff. The reason for the variable effect of puffs is not well known, but multiple actuations may influence the overall aerodynamic environment and drug flow within the chamber.

There is clear evidence that better drug delivery can be achieved by inhaling each dose separately. Most of the guidelines are in line with the evidence, but this should be noted in every guideline (Table 2).

### Electrostatic charge and detergents have variable effects on drug VHC delivery

Static electricity is an imbalance of electric charges within or on the surface of a material. Materials that have a low electrical resistance are called conductive, since they allow electrons to flow easily across the surface or through the material. Conductive materials—for example aluminum and steel—are less static compared with non-conductive materials. Non-conductive materials, such as plastic or cardboard, are always prone to build up static charge. The polarity and strength of the charges differ according to the compound used, surface roughness, ambient temperature, rinsing, and other properties. In addition, anti-static coating can reduce the triboelectric effect but cannot completely diminish it. The most challenging characteristic of the triboelectric effect is unpredictability.

Many in vitro and in vivo studies have demonstrated that drug delivery from non-conductive (e.g., polycarbonate or polyester) VHCs that are prone to static electricity is typically improved by pre-washing in detergent solution and air-drying, or via other means of antistatic coating [31, 53–55]. Performance differences exist also between those plastic VHCs that are marketed by the manufacturer as antistatic (e.g. acrylonitrile butadiene styrene (ABS)) and even between conductive (e.g. metal) VHCs [19, 31, 52, 55–59]. Reducing the charge by coating the chamber surface with ionic detergent has been shown to result in an increase of 50–70% in fine particle (< 6.8 μm) delivery from a large polycarbonate VHC (Volumatic) [54] as well as from five small volume plastic (polycarbonate and ABS) and metal VHCs in vitro [51]. In another study, washing volumatic with plain water was as effective as an antistatic lining in reducing the effects of static charge on salbutamol delivery in vivo [49]. Similarly, the study by Dompeling et al. [56] found that despite their difference in static properties, plastic VHCs (Aerochamber and Volumatic) and the metal Nebuchamber were equally effective regarding the clinical efficacy of salbutamol in children with asthma. On the other hand, Csonka and Lehtimäki [52] detected a significant reduction in the performance of Babyhaler (polycarbonate) and A2A (ABS) after detergent washing. Hatley et al. [60] found that the performance of the Optichamber Diamond (ABS), when taken out of its original packaging and used for the first time, was not influenced by washing in soapy water.

The effect of static charge of VHCs has translated to variable effects on lung deposition and lung function [19, 31, 55–58]. Piérart et al. [31] found that the mean lung deposition of radiolabeled salbutamol in healthy subjects was 46% through a detergent-coated volumatic compared with 12% through an untreated static VHC. A study by Anhøj et al. [55] reported that the antistatically treated Babyhaler delivered a significantly higher lung dose than either untreated Babyhaler or AeroChamber (ABS). In the study by Dompeling et al. [56] electrostatic charge on plastic VHCs had no significant influence on the clinical efficacy of salbutamol in children with asthma. The Nebuchamber (metal), Aerochamber (ABS), and Volumatic (polycarbonate) were equally effective [56]. The in vivo study by Janssens et al. [57] involving 1-4 years old children detected significantly (*p* < 0.001) higher mean filter dose (41.7 ± 10.1%) using metal Nebuchamber compared with polycarbonate Babyhaler 26.0 ± 4.0%. Another study found no differences in specific airway resistance or FEV1 following a methacholine challenge in children administered salbutamol via an untreated Babyhaler, a detergent washed Babyhaler or a metal Nebuchamber [58].

Evidence suggests that different chamber materials have variable electrostatic properties and that VHC handling may significantly affect drug delivery. Some guidelines address the issue of VHC static charge (Table 2), but there are no specific recommendations about the ideal chamber composition and as to how and with which detergent should the VHCs be pre-treated. Future sponsor-independent studies are needed to evaluate the clinical impact of the electrostatic effect and antistatic treatment of VHCs.

## Conclusions

We found strong evidence to support several recommendations in current guidelines. Face mask should be omitted when the child can hold the mouthpiece of the VHC tightly between the lips and teeth. In addition, each puff of salbutamol should be given separately without delay, i.e., one puff per 2–5 inhalations.

Based on the systematic review, we suggest adding a specific note to the current guidelines about the impact of cooperation during drug administration. Calming the child and securing a tight face-to-mask seal is critical for successful drug delivery. Guidelines could benefit from more elaborate instructions on drug administration with valved holding chambers in preschool children (Table 3.)

**Table 3:** Recommendations on how to use a pMDI and VHC in preschool children

These are general recommendations and the nuances may vary depending on the child’s age and device model. General notes: • Always check that the VHC is intact and the valves are correctly positioned and working properly. • Face mask should be used in children younger than three years of age and for those who are unable to hold the VHC’s mouthpiece between the lips. • Tight fit and good seal between the mask and face is essential for drug delivery. Choose the correct size mask. • Crying and poor co-operation of the child during inhalation may significantly decrease pulmonary drug delivery. Invest in calming the child but administer the medication as soon as possible. • Always actuate one puff at a time into the VHC. Drug delivery step by step 1. Explain to the child, what you are about to do and why. Calm the child if he/she is agitated. 2. Position the child sitting up straight with face slightly upwards. Support the child’s body and head gently but firmly. With small children you may need extra helping hands. 3. Remove the pMDI cap. 4. Shake the pMDI vigorously five times. 5. Hold the pMDI upright and place it firmly into the VHC. 6. Keep the pMDI+VHC unit in a horizontal position and place the face mask or mouthpiece meticulously: 6.1. In case a face mask is used, make sure the face mask is of correct size. It should cover the mouth and nose comfortably. Look around the mask’s perimeter and make sure that the mask is touching the face all around and there is no leak between the mask and face. Adjust the fit if necessary. 6.2. If the mask is not needed, place the mouthpiece between the teeth and make sure the lips are tightly sealed around the mouthpiece without gaps. 7. If the child is crying or resisting, take some time to calm the child. 8. When the VHC is properly positioned and the child is inhaling calmly, actuate the pMDI once. 9. Let the child breath for at least five breathing cycles before you remove the VHC. 10. If additional doses are needed, repeat the whole process from step 4. onwards.	

Although some guidelines address the issue of VHC static charge, there is insufficient data to make recommendations on ideal chamber material and antistatic treatment. Published data suggest that different VHCs and their masks cannot be assumed to be equally effective in drug delivery for bronchodilator administration, but there is not enough evidence to make specific recommendations on the most reliable and effective VHC delivery system. More research is needed about the optimal choice of pMDI+VHC combination for children at different age groups and with different respiratory patterns.

## Data Availability

If requested.

## Abbreviations

ABS: Acrylonitrile butadiene styrene
ER: Emergency room
PIFR: Peak inspiratory flow rate
pMDI: Pressurized metered dose inhalers
RR: Respiratory rate
VHC: Valved holding chambers
Vt: Tidal volume

## Code availability

N/A
